# The Cummings' Patent

**Published:** 1872-03

**Authors:** 


*The Cummings' Patent.*—To the question, "Does S. S. White hold any stock in the Goodyear Dental Vulcanite Company, or is he interested, pecuniarily or otherwise, in the success of the Goodyear or Cumming's patents?" Dr. White replies as follows:

"I take pleasure in saying that I am not, and that I never have been, a stockholder in the Goodyear or other Vulcanite Company, by whatsoever name called; that I am not, and never have been pecuniarily interested to the extent of a single dollar in the Goodyear, Cumming's or any other patent for the application of rubber as a base for artificial teeth; nor have I any interest in any of the materials for base in the market—celluloid included—except as a dealer."

And the following is the answer to the statement that Josiah Bacon owns the celluloid base, taken from the *Cosmos*:

"Josiah Bacon does not own the celluloid base, nor any of the patents under which it is made, sold, and used ; nor has he any interest in said base. or patents, or any portion thereof, in any manner whatsoever.—I Smith Hyatt, *Secretary Albany Dental Plate Company*.

The following communication to the *Cosmos* will throw some light upon the subject of the Cumming's patent :

"The importance of the appeal now pending in the Supreme Court of the United States, instituted to declare this patent null and void, cannot be overestimated. Considering that of the twelve thousand dentists in the United States, at least one-half, in their practice, use hard rubber, or vulcanite, for dental plates, and estimating the royalty at $75, we would have an annual tax on the profession of $450,C00, and this when the patent is in litigation. How much will it be when Josiah Bacon has a decree of the Supreme Court of the United States sustaining the patent ?

The case of the Goodyear Dental Vulcanite Company *vs.* Benoni E. Gardiner has been selected by the dental profession as a test case, and, if successful, will relieve the profession from all future exactions.

On tho 6th of May, 1872, the hard rubber patent of Nelson Goodyear will expire by its own limitation, and thenceforth Bacon must rely solely upon the Cumming's patent to tax and drain the profession, and they must now present a united front, if they expect to succeed.

A short statement of the claim of Cummings for his letters patent, his mode of obtaining them. and the defenses now presented, will enable the reader to judge of the probability of success in this appeal.

Cummings claims that he is the original inventor of the application of hard rubber for dental plates, and his whole claim hangs on the truth of this assertion. He filed a caveat in 1852, in which he states that he has discovered such an application, and wants time to perfect his invention. He applied for a patent in 1856; it is rejected. He appeals to the Board of Examiners, who reject it, and the Commissioner affirms the rejection in February, 1856. He does nothing until in 1859, when he attempts to withdraw his application, which is refused, because it has been finally rejected, when he again lies still until 1864, when he makes a new application before a new Commissioner, and obtains a patent.

The defenses, all of which are proved in the Gardiner case, are many. We will give a few :

First. Cummings was not the first one to apply vulcanite to dentistry. Perhaps, if he had discovered a *new process* of applying this, he might have properly claimed a patent ; but he claimed

*no* process, merely the bare application of this substance as a basis for dental plates.

This application was the subject of letters to Dr. Truman, in England, in 1848, who, at the World's Fair, in London, 1857, received the highest medals for his plates, which were then eulogized by the Jury of Examination for their perfect workmanship and utility. In 1850, letters patent were granted in Paris. In the year 1851, Dr. De La Barre read a long article before the Academy of Sciences of France, giving the details of his method of using it. Dr. Evans had used it in Paris in 1848, and made several sets of teeth for Goodyear in 1851. As early as 1845 Dr. Berend, of Liverpool, was using it, and Dr. John Lovejoy was doing the same thing in New York city the same year. It was described in the various journals of Europe and the United States. Cummings claims in his cavant, in 1852, that he had just discovered his application, but has not perfected it, showing that he could not then do it himself.

Secondly. It was shown that Cummings himself did not make a dental plate even up to the date of his patent. He was obliged to obtain the plates he used in his application for a patent of one Robert Haering, Dr. Mallett, of New Haven, aud Wm. A. Bevens, a workman in Goodyear's shop, in New Haven.

Third. His original patent was for an application, and without disclosing any process to manufacture a plate. It is a familiar principle of patent law that a known article or substance, applied to a well-known manufacture or use, is not the subject of letters patent. It is not patentable to merely apply the known article to a new use ; and this is all Cummings did in his first patent.

Accordingly in a few months he surrendered his patent, and obtained a re-issue, which, for the first time detailed any *process* by which such plate could be made, and now they are claiming that Cummings invented this *new process.*

Fourth. That this *process* was one well known to dentists as early as in 1856, and many were practicing it throughout the United States. In fact, Cummings was almost the only *one who* was not familiar with this process years prior to his letters patent.

Fifth. Had Cummings been the inventor and discoverer both of the application and this *process*, he clearly would not have abandoned it to the public. For eight years after the rejection of his application he allowed all the profession to use it, made no claim to any rights under it, but acquiesced, and announced to them that he had tried it and failed.

When the Hard Rubber Company sold licenses for the use of rubber, *he took a license* from them. When his assistant wished to use it in 1860 he could not teach him how to make it, and employed and paid a young man to instruct him in the art. Unquestionably the idea of getting the patent in 1864 had never occurred to him as late as 1862, and probably dates from his acquaintance with Josiah Bacon.

To claim that he did not abandon to the public necessities the proposition that he *could* not abandon it, as he did all he could to show such abandonment.

Sixth. The re-issue was a different invention from the original patent, which would render it void. If the first patent was merely for the application, and this was not patentable, then the re-issue conld not be so by inserting in it a new and patented claim. A patent cannot be issued for an invention which the inventor has suffered to go into public use for two years; and he must apply for his patent within two years after making his invention. If his original invention of 1856 was for a *process*, then his re-issue of 1865 was practically a new invention, and had been sufficient to go into public use more than two years, and hence was void.

Seventh. Two other objections, technical in their nature, but fatal if well taken, are that the original company, the Dental Vulcanite Company, incorporated under the Massachusetts law, and the present company, the Goodyear Dental Vulcanite Company, incorporated under the New York manufacturing act, are each incorporated merely and solely for *manufacturing purposes*, and any business they attempt beyond those mentioned in this charter is *ultra viris.* Hence they would have no legal capacity to enter into the business of selling licenses to dentists; and the re-issued patent, which is the only one they now own, being issued to a corporation who had no capacity to receive it, is void. For, if the corporation cannot receive it, they cannot use it, or even sell it.

There are other points relied upon; but the above represent these most important, and either of which is believed to be fatal to the claims of the company and Bacon.

All the facts upon which the above are based were fully proved, and none were *contradicted.*

Unquestionably the fact of abandonment is the most palpable one, and, coupled with the refusal to call, or suffer to be called, Cummings as a witness, to prove by him his intention to claim his invention during the eight years abandonment, and his failure to deny his ignorance of the *process*, seems to leave the Supreme Court no option but to refuse Judge Clifford, and relieve the profession from this incubus."

